# Parental ages and levels of DNA methylation in the newborn are correlated

**DOI:** 10.1186/1471-2350-12-47

**Published:** 2011-03-31

**Authors:** Ronald M Adkins, Fridtjof Thomas, Frances A Tylavsky, Julia Krushkal

**Affiliations:** 1 Department of Pediatrics, University of Tennessee Health Science Center, Memphis, TN 38103, USA; 2 Department of Preventive Medicine, University of Tennessee Health Science Center, Memphis, TN 38163, USA

## Abstract

**Background:**

Changes in DNA methylation patterns with age frequently have been observed and implicated in the normal aging process and its associated increasing risk of disease, particularly cancer. Additionally, the offspring of older parents are at significantly increased risk of cancer, diabetes, and neurodevelopmental disorders. Only a proportion of these increased risks among the children of older parents can be attributed to nondisjunction and chromosomal rearrangements.

**Results:**

Using a genome-wide survey of 27,578 CpG dinucleotides in a cohort of 168 newborns, we examined the relationship between DNA methylation in newborns and a variety of parental and newborn traits. We found that methylation levels of 144 CpGs belonging to 142 genes were significantly correlated with maternal age. A weaker correlation was observed with paternal age. Among these genes, processes related to cancer were over-represented, as were functions related to neurological regulation, glucose/carbohydrate metabolism, nucleocytoplasmic transport, and transcriptional regulation. CpGs exhibiting gender differences in methylation were overwhelmingly located on the X chromosome, although a small subset of autosomal CpGs were found in genes previously shown to exhibit gender-specific differences in methylation levels.

**Conclusions:**

These results indicate that there are differences in CpG methylation levels at birth that are related to parental age and that could influence disease risk in childhood and throughout life.

## Background

DNA methylation is a normal, heritable epigenetic modification that down-regulates the expression of approximately 1/3 of human genes [1-3] and is key to the allele-specific imprinting of genes [4]. DNA methylation also plays an important role in disease. For example, overall DNA hypomethylation accompanied by gene-specific hypermethylation is a hallmark of cancer [5]. Additionally, shifts in DNA methylation patterns appear to be involved in the normal aging process and increase in disease susceptibility [6]. Indeed, there is ample evidence for characteristic changes in the patterns of DNA methylation with age.

The earliest data supporting progressive changes in DNA methylation patterns with age came from global studies of blood that demonstrated lower levels of methylation in older individuals [7] and greater differences in methylation in older monozygotic twins [8]. Christensen et al. [9] examined 217 tissues sampled from a range of non-diseased solid tissues and blood. In solid tissues the general trend was towards methylation increasing with age in CpG islands and decreasing outside of islands. However, in 85 blood samples the predominant pattern was a decrease in DNA methylation with age that was independent of CpG island status. In an analysis of 280 CpGs measured from whole blood from 46 monozygotic and dizygotic twin pairs and 96 singleton adults, Boks et al. [10] found both increases (60%) and decreases in methylation with age among the 100 most significant results. As previously observed, levels of methylation for X-linked loci were higher among females. Heritability estimates for methylation levels for 25 significant (p ≤ 10^-3^) CpGs ranged from 0.57 - 0.94. Similarly, in whole blood samples from 93 females using Illumina's Humanmethylation27 BeadChip (the same microarray as used in this study) Rakyan et al. [11] found 213 CpGs whose methylation levels positively correlated with age ("hyper-aDMRs") and 147 that negatively correlated. In a replication cohort of 20 females and 5 males, 60% (131) of CpGs whose methylation increased with age replicated in CD14^+ ^monocytes and CD4^+ ^T-cells, while the CpGs whose methylation decreased replicated in the T-cells but not monocytes. Rakyan et al. suggested that the lack of replication in the monocytes was due to changes in cell composition in blood with age. Only the hyper-aDMRs were studied in buccal cells from ten individuals, where they showed high replication. A larger study [12] (N = 197, 21 - 55 years old) of DNA isolated from the saliva of alcohol abusers using the same Illumina array as the one reported here found 85 CpGs significantly correlated (including 29 negatively) with age after Bonferroni correction. In another study using the same array for blood from 261 postmenopausal women, Teschendorff et al. [13] found significant hypermethylation of 226 CpGs and hypomethylation of 363 CpGs with age. Replication results were not presented for all significant CpGs, but focusing on only those mapped to polycomb group proteins the 64 genes with hypermethylated sites replicated in other tissues and individuals, while the 11 hypomethylated genes generally did not. Once again, this could be due to changes in blood cell composition with age.

The studies summarized above investigated the relationship between age and DNA methylation levels in a cross-sectional sample of individuals at a fixed point in time. Studies that have examined changes in DNA methylation levels in the same individuals over time have revealed similar results. Bollati et al. [14] studied levels of DNA methylation in *Alu *and *LINE-1 *repetitive elements in serial blood samples taken up to eight years apart from 718 elderly Boston individuals (55 - 92 years old). They found a significant correlation between age and DNA methylation in both types of elements and significant declines in methylation in *Alu *elements in the same individual over time. Similarly, Bjornsson et al. [15] studied changes in DNA methylation in blood in the same individuals over a span of greater than 10 years. Although results were not presented for all families or as summaries across all families, the 50 largest changes in blood mostly appeared to be decreases both inside and outside of CpG islands. After excluding one family that exhibited unusually consistent changes in DNA methylation over time, Bjornsson et al. estimated a heritability for DNA methylation change over time of 0.743.

There is typically a very high correlation between the ages and social/environmental exposures of parents that makes it difficult to disentangle the relative roles of maternal and paternal ages in adverse outcomes in their offspring. Generally, very large epidemiological studies are required that include a statistically meaningful subset of parents with discordances in age. Based on such studies, both maternal and paternal age correlate with elevated risks of complications during pregnancy and complex disorders in the offspring, such as intrauterine growth retardation, altered placental morphology, preeclampsia, and preterm labor [16]. Although the connection is controversial in some cases, the offspring of older parents have increased risks of various disorders, such as type 1 diabetes [17], obsessive-compulsive disorders, autism [18], schizophrenia [19], bipolar disorder [20], stuttering [21], hematologic cancers [22], and the more common childhood cancers [23-29]. Furthermore, daughters of older women have an increased risk of breast cancer [30], and the sons of older fathers have an increased risk of prostate cancer [31]. The role of epigenetics in these associations is only beginning to be explored. The only recent recognition of a possibly large role of epigenetics may be partially attributed to assumptions that each generation begins with a "renewed" epigenome. After fertilization the maternal and paternal genomes undergo a rapid demethylation of most of the genome followed by remethylation. Naively, this suggests that each generation begins with a refreshed epigenome that, barring gross environmental insults or altered dietary intakes [32], is relatively immune to effects from the previous generation [33,34]. Here we report the results of genome-wide DNA methylation profiling of 168 newborns that indicates that there is a general trend towards hypomethylation of CpG islands in newborn blood cells with increasing parental age. It is possible that this trend provides a partial explanation for the association between increased parental age and adverse outcomes in offspring.

## Results

### Final characteristics of the data set and population

Across all 168 newborns, two CpGs exceeded a median detection p value of 10^-6 ^and were excluded from analysis. The final data set was comprised of 27,576 CpG dinucleotides. An average of 184 ± 498 (range 0 - 2,989) CpGs per newborn exceeded the threshold detection p value of ≤ 10^-3^, and those specific CpGs within individual newborns were dropped from analyses.

The distribution of maternal ages was skewed towards younger mothers, with 41% (N = 69) being younger than 26 and the proportion diminishing with increasing age (Table 1; 26-30 yrs, 33%, N = 55; 31-35 yrs, 20%, N = 34; 36-39 yrs, 6%, N = 10). Most mothers were classified as normal weight or overweight at the time of recruitment according to body mass index (BMI), with 5.4% (N = 9) mothers being underweight (BMI < 18.5), 46% (N = 77) normal (BMI = 18.5 - 24.9), 20% (N = 34) overweight (BMI = 25 - 29.9), and 29% (N = 48) obese (BMI > 29.9). The majority of newborns were born at term (≥ 37 weeks; N = 158), while seven were near-term (35 - 36 weeks) and three were preterm (32 - 33 weeks). Birth weights ranged from 1.2 to 5 Kg, with the lower end of the range primarily due to the small number of preterm newborns. There were slightly more male (N = 88, 52%) than female newborns. There was no difference in the average age of mothers giving birth to female or male newborns (t test, p = 0.91). The age was reported for 89 fathers, which averaged 31.6 years. Unlike the maternal ages, the distribution of paternal ages was normally distributed (Shapiro-Wilk W test, p = 0.74; 18 - 25 years, 20%, N = 18; 26 - 30 years, 21%, N = 19; 31 - 35 years, 29%, N = 26; 36 - 40 years; 21%, N = 19; 41 - 50 years, 8%, N = 7).

**Table 1:** Characteristics of the participants (n = 168).

Variable	Mean (SD)	Range
Race (Maternal)		
African-American (N)	92	
Caucasian (N)	70	
Other, Multiracial (N)	6	
Mothers		
Age (years)	27.0 (5.1)	18 - 39
Parity	1.1 (1.2)	0 - 5
BMI (Kg/m^2^)	27.0 (7.3)	15.3 - 54.9
Smoke during pregnancy (N)	15	
Paternal age (N = 89)	31.6 (7.1)	18 - 50
Newborns		
Gestational age (weeks)	39.0 (1.3)	32 - 41
Female (N)	80	
Birth weight (Kg)	3.3	1.2 - 5.0

### Relationship between newborn CpG methylation levels and maternal/newborn variables

No CpG displayed a significant relationship between methylation level and gestational age, parity, maternal BMI, birth weight, or to self-declared smoking during pregnancy. Across all chromosomes, the levels of methylation at 144 CpG loci located in 140 autosomal (2 each in *ARFGAP3 *and *MEA1*) and 2 X-linked genes exhibited genome-wide significant correlations with maternal age (Additional File 1, Table S1). Relative to other loci, imprinted genes generally maintain a stringent pattern of allele-specific methylation in a parent of origin manner. Nevertheless, a single CpG in each of two imprinted genes (*KCNQ1 *and *DIRAS3*) were the third- and eighteen-ranked sites (rho = -0.41 and -0.40, respectively) correlated with maternal age. The level of methylation at all but 9 of the 144 genome-wide significant CpGs was negatively correlated with maternal age. With the exception of six genes, at least two CpGs are interrogated in each of the 142 genes containing genome-wide significant CpG probes. To determine if the pattern we observe for the genome-wide significant CpGs is reflected by another CpG in the same gene, we examined the direction and significance of the Spearman rho for the CpG with the next lowest p value. Of the 136 genes represented by multiple probes, 95 of the second-ranked CpGs exhibited a correlation in the same direction as the top-ranked CpG, of which 53 were significant at a nominal p value ≤ 0.05 (Additional File 2, Table S2). The levels of methylation of these 53 pairs of CpGs are significantly correlated (Spearman's rho = 0.26 - 0.82, p = 6.3 × 10^-4 ^- 2.1 × 10^-42^), except the pair in the *GORASP1 *gene (rho = 0.07, p = 0.39).

**Figure 1:**
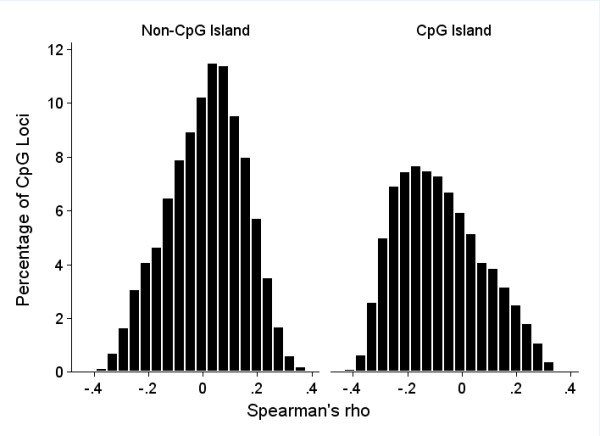
**Distribution of the Spearman rank correlation rho statistics between maternal age and newborn CpG methylation**. Using the annotation of the Humanmethylation27 array provided by Illumina Inc., the distribution of rho statistics was plotted for CpG dinucleotides outside of CpG islands (left) and inside islands (right).

Of the 144 CpGs exhibiting a significant correlation with maternal age, all but 8 (5.6%) are located in CpG islands. By contrast, 7,568 (27.45%) of the CpGs in the total data set are outside of a CpG island. If there were no relationship between the CpG island status of each probe and its correlation with maternal age, we would expect 39-40 of the significantly correlated CpGs to be outside of an island. Therefore, there is a significant (binomial test, p = 1.6 × 10^-11^) over-representation of CpG island sites among the significant correlations. Of the nine significantly positive correlations between maternal age and newborn CpG methylation, four are outside of a CpG island. Indeed, overall there appears to be a different relationship between maternal age and levels of methylation of CpGs located inside and outside of CpG islands (Figure 1). Outside of CpG islands, the distribution of Spearman rho statistics is slightly shifted towards positive correlations (median = 0.02), while inside CpG islands the correlations are predominantly negative (median = -0.1).

Of the 834 CpG loci (501 genes) whose methylation levels are significantly associated with newborn gender, all but 16 (14 genes) are located on the X chromosome, and these associations are presumably due to a systematic difference between males and females in the average level of DNA methylation on the X [35]. Six (*C6orf68*, *TLE1*, *GLUD1*, *NUPL1*, *FLJ20582*, *FLJ43276*) of the fourteen autosomal loci exhibiting gender-specific differences in methylation levels were also among the eleven loci found to exhibit genome-wide significant gender differences in adult saliva DNA based on the same Illumina array in a study by Liu et al. [12]. Additionally, and consistent with our observation, Philibert et al. [36] demonstrated higher methylation of the *SLC6A4 *gene, posited to be important in several psychiatric and behavioral traits, in adult females. Combined, these results indicate that these gender-specific differences in methylation of a small number of autosomal genes are replicable, present in multiple tissues, and established by the time of birth. Other than zona pellucida-binding protein 2 (*ZPBP2*), none of the 14 autosomal genes has an obvious function related to gender. The gender difference in X chromosome methylation could confound the ability to detect a relationship to maternal age in analyses that include both genders. When analyses were restricted to individual genders, the DNA methylation level of one X-linked probe (cg08800033 at *CETN2*) was genome-wide significant in male newborns, and none were significant in females. If a less stringent Bonferroni cut-off (p = 4.6 × 10^-5^) is employed based on the 1,085 CpGs located on the X, then three X-linked loci (cg08800033 at *CETN2*, cg14570389 at *RAP2C*, and cg17880859 at *RBBP7*) in males and one (cg06350107 at OTC) in females is significant. In light of these results and the expected decrease in power to detect a trend in single genders, it is possible that the levels of methylation of a small subset of X-linked CpGs exhibit a correlation with maternal age in a gender-specific manner. However, the support for this is weak, and we did not further examine gender-specific patterns for X-linked CpGs with respect to maternal age.

### Replication of maternal age correlation by pyrosequencing

For 92 newborn DNA samples, the correlation between maternal age and DNA methylation was reassessed using pyrosequencing for three CpGs. These CpGs were chosen based on their different levels of methylation and the correlation of the methylation levels with maternal age. Those three sites and their levels of methylation and correlation with maternal age based on Illumina data were cg09118625 (47 - 82%, rho = -0.38, p = -.0002), cg18984151 (10 - 25%, rho = -0.34, p = 0.001), and cg21576698 (3 - 23%, rho = -0.31, p = 0.003). Based on pyrosequencing, the correlations with maternal age were replicated (cg09118625, rho = -0.27, p = 0.009; cg18984151, rho = -0.28, p = 0.007; cg21576698, rho = -0.42, p = 3.6 × 10^-5^). However, while the inferred levels of methylation of cg18984151 from pyrosequencing (12 - 23%) were very similar to and highly correlated with the values from the array (rho = 0.75, p = 4.6 × 10^-18^), the correlations for the other sites were strong but the inferred methylation levels shifted downwards (cg09118625, 39 - 48%, rho = 0.73, p = 2.4 × 10^-16^; cg21576698, 0 - 1.4%, rho = 0.28, p = 0.008). Altogether these results indicate that the correlation between maternal age and newborn DNA methylation is replicable, but that the absolute methylation values derived from the Humanmethylation27 array for some sites may be biased indicators of the true values and may overestimate the true level of inter-individual variation in methylation levels.

### Relative strength of the correlation of maternal vs. paternal age with newborn CpG methylation

**Figure 2:**
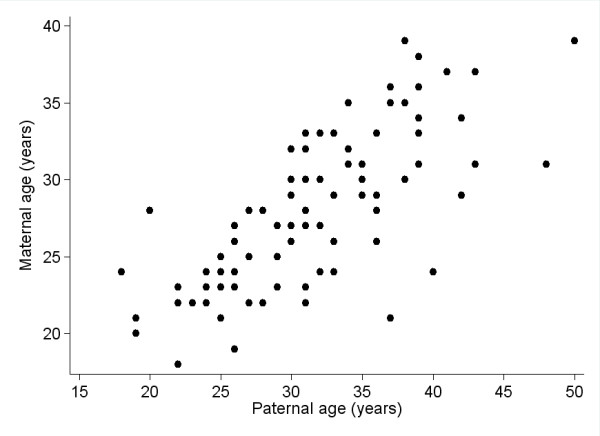
**Scatterplot comparison of maternal and paternal ages among 89 newborns for which the age of both parents was known**.

The ages of the mothers and fathers were highly correlated in our sample (Figure 2; Spearman rho = 0.75, p = 2 × 10^-17^). As a consequence, it would be extremely difficult to distinguish between the effects of maternal and paternal age. This is particularly the case given that there are only 31 cases of an age difference ≥ 5 years and 7 cases of an age difference ≥ 10 years, providing little statistical power to examine these subsets as a unique category. The relative strength of the relationship between CpG methylation and parental ages can be qualitatively assessed by examining the relative magnitude of the correlation between methylation levels and the ages of the 89 mothers and fathers for which we have the ages of both. With only 89 observations, the power to achieve genome-wide significance is reduced. For paternal age, there are 6 correlations with a p value ≤ 10^-4 ^(84 at p ≤ 10^-3^), while for maternal age there is one CpG significant at the genome-wide level (cg00231644 in *TUBB2A*, p = 1.54 × 10^-6^), 7 additional CpGs with p ≤ 10^-5^, and a total of 106 with p ≤ 10^-4^. Therefore, it appears that the relationship of newborn CpG methylation with maternal age is stronger and involves a larger number of sites than does that with paternal age.

### Role of Population Stratification or Sequence Polymorphism

**Figure 3:**
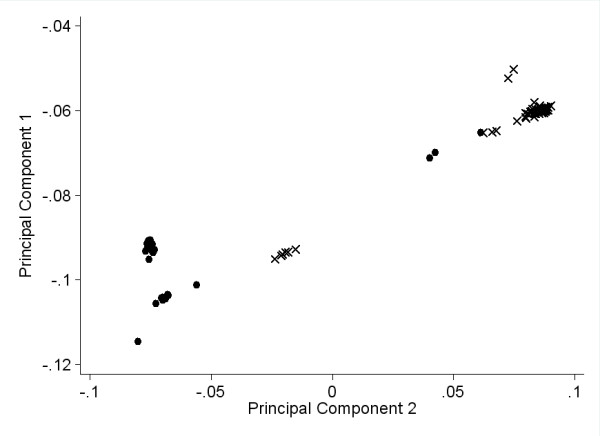
**Scatterplot of newborns along the first and second principal components calculated from genome-wide single nucleotide polymorphism data**. Maternally-declared Caucasian newborns are represented by circles and African-American newborns by X's.

We have previously demonstrated extensive differences in DNA methylation levels between African-Americans and Caucasians [37]. Therefore, our observed correlations between maternal age and newborn DNA methylation levels could be confounded by these racial differences, particularly given that the African-American mothers are generally younger than the Caucasian mothers (p = 0.0001, Wilcoxon rank-sum test). Based on plots of the first two principal components using genome-wide single nucleotide polymorphism (SNP) data from a subset of newborns (60 African-American, 71 Caucasian; Figure 3), two rather distinct clusters of individuals defined by both principal components are clear. One cluster is composed of 57 maternally-declared African-American and 3 Caucasian newborns, and the other contains 6 African-American and 65 Caucasian newborns. As expected based on the significant difference in maternal ages between the two races, the first genetic principal component is significantly correlated with maternal age (rho = -0.28, p = 0.0001), reinforcing the possibility for confounding of racial and maternal age effects. However, within each of the two clusters, the correlation between the first principal component and maternal age was not significant (p > 0.2), suggesting that analyses within these clusters of modest genetic homogeneity would be much less subject to confounding of racial, genetic, and maternal age effects.

Within each cluster defined by genetic principal components, correlation analysis was performed between maternal age and DNA methylation at each CpG. Given the reduction in the sample size within each cluster to less than half the total sample size, it is not surprising that only one CpG achieves a genomewide significant correlation with maternal age. Nevertheless, for the purposes of evaluating the possibility of confounding between maternal age and race, it is more relevant to compare the trends between the genetically distinct clusters of newborns. A correlation analysis of the Spearman rho statistics between the two clusters is highly significant (rho = 0.39, p < 10^-14^; using newborns declared as only one race, N = 162, rho = 0.41, p < 10^-14^), indicating that similar trends exist in both groups. Restricting attention to rho statistics with a magnitude ≥ 0.2 (roughly p ≤ 0.1), indicating a relatively strong trend, there are 2,584 CpGs which achieve this level in both groups. Of these, only 175 (7%) have rho statistics with opposite signs, meaning strong trends in the opposite directions. Overall, these results indicate that correlations between maternal age and newborn CpG methylation are similar in groups defined by race or genetic principal components and that confounding by racial differences in DNA methylation levels or average maternal age is not a major influence.

Although overall racial differences in DNA methylation levels do not appear to be confounding our observation of correlations between maternal age and newborn DNA methylation, a more subtle confounding could arise from the presence of sequence polymorphisms, particularly SNPs, within the regions targeted by probes on the Humanmethylation27 array whose allele frequencies differ between the races and which could affect the hybridization of DNA to the array. Using the UCSC Genome Browser http://genome.ucsc.edu we examined a window of 50 nucleotides upstream and downstream of each of the 144 genomewide significant CpGs correlated with maternal age. Of the 144 significant CpGs, 60 are within 50 nucleotides of at least one polymorphism listed within dbSNP http://www.ncbi.nlm.nih.gov/SNP/. Therefore, up to 42% of our genomewide significant correlations with maternal age could be artifacts caused by known polymorphisms in the human genome. Given that we used a rather liberal criterion for defining potential sequence effects and did not directly determine if the probes on the Illumina arrays physically overlap these polymorphisms, the number of potential artifacts due to altered hybridization is undoubtedly lower than 42%.

### Functional Clusters and Disease Processes

Among the 1,494 CpGs correlated with maternal age at a false discovery rate of 6.69 × 10^-4 ^(see Methods), 1,423 genes are represented, including 12 imprinted loci (*KCNQ1*, *PRIM2*, *PLAGL1*, *GRB10*, *H19*, *IGF2*, *CDKN1C*, *WT1*, *TCEB3C*, *PEG3*, *GNAS*, *ZNF331*). Cancer was the single disease category (1.43-fold enrichment, p = 3.42 × 10^-5^) significantly over-represented based on the presence of 96 genes associated with its etiology or progression (Additional File 3, Table S3). Clustering of those 1,423 genes based on ontology resulted in 14 significantly over-represented functional categories (Table 2; Additional File 4, Table S4). The principal functions represented by these categories were mesodermal development, metabolic regulation, nucleo-cytoplasmic transport, and transcriptional regulation.

**Table 2:** Functional clusters of 1,493 CpGs (1,423 genes) with strongest correlations between levels of methylation and maternal age according to DAVID.

Terms	# Genes	Minimum DAVID p Value	Enrichment Score
Mesenchymal cell development/differentiation	11	0.003	2.53
Formation of mesoderm/primary germ layer	9	0.003	2.34
Neurological regulation	19	0.008	1.83
Regulation of glucose/carbohydrate metabolic process	8	0.01	1.83
Unsaturated fatty/icosanoid metabolism/biosynthesis	8	0.005	1.71
Negative regulation nucleocytoplasmic protein/transcription factor transport	5	0.006	1.70
Regulation nucleocytoplasmic protein/transcription factor transport	10	0.01	1.60
Limb morphogenesis	14	0.02	1.57
Phosphopantetheine-binding, acyl carrier activity	3	0.01	1.53
MADS-box	3	0.04	1.40
Helix-loop-helix	14	0.03	1.37
Positive regulation of glucose/carbohydrate metabolism	5	0.04	1.33
Negative regulation of phosphorylation	8	0.04	1.33
Regulation of transmembrane transport	6	0.03	1.32

### Relationship between maternal age and newborn DNA methylation in blood cell type signature genes

Of 852 loci with official gene symbols whose expression levels are signatures of blood cell types [38], we found 658 that were assayed by the Humanmethylation27 array. These 658 genes are represented by 1,273 CpG probes on the Illumina array (Additional File 5, Table S5). The first principal component based on the methylation levels of these probes among the 168 newborns explained 97.9% of the variation. Both visually and based on Spearman correlation (rho = 0.07, p = 0.34), there was no apparent relationship between the principal component representing combined blood cell signature gene methylation and maternal age. Of those 1,273 individual probes, 7 (0.55%; within genes *C19orf6*, *GM2A*, *SEPX1*, *RPL27*, *CTSZ*, *PGAM1*, *PLXDC1*) were among those significantly correlated with maternal age. Given that this proportion is nearly identical to the proportion of CpG probes genome-wide showing significant correlation with maternal age (0.52%; P(7/1,273 ≥ 0.52%) = 0.49, binomial test), blood cell type signature genes do not appear to be enriched among the significant CpG probes, thereby providing indirect evidence against changes in umbilical blood cell composition with maternal age.

## Discussion

To the extent of our knowledge, this is the first demonstration of a trans-generational effect of parental, primarily maternal, age on the levels of DNA methylation in the next generation at birth. Clusters of CpG dinucleotides, or CpG islands, are disproportionately located in the upstream, regulatory regions of genes and typically exhibit lower levels of methylation than do non-island CpGs. In this sense the trend we observed towards decreased methylation of newborn CpG islands with increasing parental age represents an accentuation of a normal pattern of DNA methylation. In this context, the question becomes whether these changes are related to disease or other phenotypic outcomes in the offspring. As was noted earlier, the children of older parents have increased risks of type 1 diabetes, neurodevelopmental disorders, and cancer. Therefore, it is noteworthy that cancer was the disease category over-represented by genes whose methylation strongly correlated with maternal age. A survey of Online Mendelian Inheritance in Man (OMIM) [39] for the 142 genes that achieved genome-wide significance with age in our sample reveals several genes with known functional or molecular associations with cancer, obesity, and diabetes (Table 3). This is suggestive of a possible role of maternal age-associated shifts in newborn methylation in the increased risks of these outcomes. However, it must be emphasized that the associations listed in OMIM are almost exclusively due to genetic defects in these genes, while hypomethylation of CpGs, particularly in CpG islands, is expected to result in increased expression of these genes, rather than repression.

**Table 3:** Diseases and functions reported to be associated with genes exhibiting a significant correlation between methylation and maternal age.

Gene	Phenotype/Function	OMIM Reference
KCNQ1	Long QT Syndrome 1; Jervell and Lange-Nielsen Syndrome 1; Atrial fibrillation, familial 3; short QT Syndrome	192500 220400 607554 609621
TUBB2A	Polymicrogyria, asymmetric	610031
RAD51	Breast cancer, familial	114480
C19orf6	Ovarian, breast, pancreatic and colorectal cancer expression	611011
RAD54L	Adenocarcinoma, colonic, somatic; Lymphoma, non-Hodgkin; Breast cancer, invasive ductal	603615
ACADS	SCAD deficiency	201470
GM2A	Gangliosidase, AB variant	272750
STAT5B	Growth hormone insensitivity with immunodeficiency	245590
RET	Multiple endocrine neoplasia, type IIA; Hirschprung disease; Central hypoventilation syndrome; renal agenesis	142623 209880 191830
ZFYVE26	Spastic paraplegia 15, autosomal recessive	270700
RBBP9	Transformation to cancer	602908
MRPS22	Combined oxidative phosphorylation deficiency 5	611719
MYC	Burkitt lymphoma	113970
RFXDC1	Diabetes, neonatal, with pancreatic hypoplasia, intestinal atresia and gallbladder aplasia or hypoplasia	601346
MYST1	Histone acetyltransferase	609912
RASSF1	Tumor suppressor	605082
ZBT16	Skeletal defects, genital hypoplasia, and mental retardation	612447
ACTN4	Focal glomerulosclerosis 1	603278
NR0B2	Obesity, mild, early-onset	604630
ALX4	Parietal foramina 2	609597

According to DAVID [40,41], there were 14 functions enriched among the 1,493 genes with the strongest correlations between maternal age and newborn DNA methylation. The category with the second greatest enrichment was mesodermal development, and this is not surprising given that white blood cells originate from the mesodermal layer. The third greatest enrichment was for functions related to neurological regulation, including strong candidate genes for psychiatric disorders such as *SLC6A1*, *GNAI3*, *PTGS2*, and *CTNND2*. Given the association between increasing parental age and schizophrenia, bipolar disorder and autism in offspring, studies of shifts in the methylation of these genes in offspring of older parents are warranted. Similarly, the risk of diabetes appears to be elevated in the children of older parents, and functions related to glucose and carbohydrate metabolism are enriched among the genes whose methylation is correlated with maternal age, including such candidate genes for obesity and diabetes as *IRS1*, *IRS2*, *DUSP12*, *DYRK2*, and *ARNT*.

Multiple reports have documented significant changes in DNA methylation patterns with age in adults, both as a genome-wide average and at specific nucleotide sites. The question arises as to whether these adult patterns mirror those we observed in newborns, which would imply that the same processes are operating both in adult somatic tissues and in either gametogenesis or embryonic development. Of the 589 CpGs with significant correlation with age in adults discovered by Teschendorff et al. [13], only one (in *PLAT*) achieved genome-wide significance among our newborns. Similarly, of the 131 CpGs studied by Rakyan et al. [11] whose methylation levels in adults were positively correlated with age and were highly replicable, only one (in *RFXDC1*) was observed by us to have a trans-generational effect. By contrast, 30 genes overlapped between Rakyan et al. and Teschendorff et al. Among the 288 CpGs studied by Boks et al. [10], 90 loci located in 69 genes were associated with adult age at p ≤ 0.05. CpGs in two of those genes (*GAS7 *and *RYK*) were also significant in our cohort. Likewise, of the 106 CpGs in 73 genes found to be significantly related to age in blood by Christensen et al. [9], only one gene (*PLAT*) was among our significant results. With the exception of a very small number of genes, these comparisons indicate that the processes resulting in age-related trends in DNA methylation in adult somatic tissues and in newborn somatic tissues may be independent.

There are two most likely possibilities to explain the differences. First, the patterns of change in methylation in oocytes and/or spermatocytes with respect to age are different than in somatic tissue and these are reflected in shifted patterns in the newborn. This explanation can be tested by comparison of methylation patterns in somatic tissues and gametes in the same individuals with respect to age. Second, the mechanisms of establishment and/or maintenance of DNA methylation in the developing fetus could be responsive to parental age, but in a manner different than in the adult somatic tissues. This hypothesis cannot be tested by epidemiological methods but instead requires detailed molecular biological examination.

It is well-established that DNA methylation of imprinted genes is established in a parent of origin manner during development of the germ line and gametogenesis [42], while methylation of other loci is thought to be re-established after a nearly global demethylation subsequent to fertilization and uterine implantation. Given the stringency with which DNA methylation is thought to be maintained at imprinted loci, it is somewhat surprising that DNA methylation at 2 imprinted loci exhibited genomewide-wide significant correlations with maternal age and a total of 12 met the less stringent q value threshold of 6.69 × 10^-4^. However, there is evidence that the methylation patterns present in the parents are not totally erased and reset during gametogenesis and fertilization at a proportion of loci, including both imprinted and non-imprinted genes. Borgel et al. [43] identified a set of 215 genes that are methylated in the mouse very early in embryonic development and well before implantation. Of the few loci tested, all were methylated in gametes, with some shifts in those patterns being observed in preimplantation embryos. Although these observations do not address the issue of whether age-related changes in DNA methylation previously found in somatic tissues are mechanistically related to the trends we have tentatively identified to be related to maternal age, they do indicate a definite potential for trans-generational inheritance of DNA methylation patterns

A proportion of the age-related changes in DNA methylation observed in adult cohorts has been attributed to changes in blood cell composition as an individual ages. Therefore, the possibility exists that the changes in patterns of newborn DNA methylation which were observed may be at least partly due to changes in umbilical cord blood cell composition during pregnancy. However, we found no significant relationship between gestational age and CpG methylation in newborn cord blood, and therefore the gestational age of the fetus is an unlikely explanation for these differences. There is no obvious mechanism by which the mother's (or father's) age would shift cord blood cell composition. We did not directly assess the relative proportion of cell types in our cord blood samples. In our examination of the methylation patterns among genes whose expression levels are characteristic of major blood cell types, we found no obvious relationship to maternal age or over-representation of these genes among the significant results. Although this does not disprove that changes in blood cell composition explain all or part of our results, these findings are not consistent with substantial differences in blood cell composition or the regulation via methylation of the genes that are signatures of major cell subtypes.

There is the obvious question as to whether the changes in DNA methylation patterns we observed in umbilical cord blood are representative of patterns in other tissues of the newborn. It is widely accepted that diverging patterns of methylation at individual loci are a key aspect of cellular differentiation during development, and as a consequence levels of methylation at a subset of loci between two different tissue types will differ. However, there is also evidence of similarities in patterns of methylation across tissue types. For example, methylation levels of *IGF2 *and *ER-α *in peripheral blood mirror those of colon tissue [44,45]. Additionally, comparisons among multiple tissues from multiple autopsy subjects reveal individual-specific and locus-specific patterns of DNA methylation but a high degree of similarity among tissues within the same individual [46]. Likewise, within an individual there is similarity in methylation patterns between blood and buccal cells [47]. Given that these similarities are shared among tissues with different embryonic origins, this suggests that the similarities are established early in development and are fairly persistent throughout life.

## Conclusions

At a subset of loci, levels of DNA methylation in umbilical cord blood are strongly correlated with maternal, and to a lesser extent paternal, age. This correlation is predominantly negative and disproportionately occurs within CpG islands. Genes associated with oncogenesis and cancer progression are significantly over-represented among the genes correlated with maternal age, and this suggests a link to known increased risks of cancer among the children of older parents. Similarly, gene functions related to neurodevelopment and neuroregulation are over-represented among the strongly correlated genes, and this may have relevance to the increasing risk of neurodevelopmental and psychiatric disorders in offspring as parental ages increase.

## Methods

### Participants and Samples

From a longitudinal study (Conditions Affecting Neurocognitive Development and Learning in Early Childhood; CANDLE) of child development from the second trimester of a singleton pregnancy until age 3 in Memphis TN, the first 168 newborns were chosen for which blood samples were available for both the mother and her newborn and for which there were no infectious or chronic illnesses in the mothers and no pregnancy complications. Newborn blood was collected from the umbilical cord at delivery. After centrifugation of whole blood, the buffy coat was collected and frozen. Subsequently, Wizard genomic DNA purification reagents (Promega Corp.) were used for DNA extraction. All demographic and phenotypic data on the mothers and newborns were abstracted from clinical records. Paternal ages were reported by the mothers via mail questionnaires and phone calls during the months after delivery of the newborn. This research was approved by the Institutional Review Board of the University of Tennessee Health Science Center, and informed consent was obtained from all mothers.

### Genome-wide measurement of DNA methylation levels

Bisulfite conversion of 750 ng of genomic DNA was performed using EZ DNA Methylation reagents (Zymo Research). Samples were then processed according to manufacturer's specifications and hybridized and scanned on the Humanmethylation27 BeadChip (Illumina Inc.) in batches of 24 samples using the Illumina BeadStation. The Humanmethylation27 BeadChip bears probes for 27,578 specific CpG dinucleotides assigned to 14,495 loci. The level of methylation of each CpG is represented by a beta value, which is calculated as the level of the fluorescence for the probe specific for 5-methylcytosine divided by the fluorescence from the probes for both the methylated and unmethylated C at that position. Consequently, the beta values span the bounded range 0 - 1. The data has been submitted to the Gene Expression omnibus data base with accession number GSE27317.

Subsequent to statistical analyses, three CpGs with differing levels of methylation from the Illumina data that also were among the top-ranked p values were chosen for validation by pyrosequencing. This work was performed by a commercial service (EpigenDx, Worcester, MA). Upon receipt of 92 DNA samples, EpigenDx performed the bisulfite conversion, designed the assays, and performed pyrosequencing with the inclusion of high and low methylation controls. For all three assays, results from control DNAs indicated complete bisulfite conversion.

### Correlation between newborn CpG methylation and both parental and newborn characteristics

Raw output files from array hybridization experiments were processed using GenomeStudio software (Illumina Inc.). This software reports detection p values for each CpG interrogated by the array, which are an indication of the ability to distinguish the target sequence from background. Beta values representing the proportion of methylation at each site and the corresponding detection p values were imported into Microsoft SQL Server 2005, where filtering of the data values was performed before statistical analyses. For each newborn, probes with detection p values ≥ 10^-3 ^were dropped from that individual. Additionally, any probe with a median detection p value ≥10^-6 ^across newborns was dropped from all individuals.

Statistical analyses were performed using SPSS version 18 (SPSS Inc.) and Stata 10 (Stata Corp.). Several variables related to the mothers, fathers and newborns could possibly be related to DNA methylation levels at individual CpG sites. Therefore, we performed statistical analyses to examine the association between each of these variables and the level of methylation at each CpG. Because the distribution of DNA methylation at many sites is not normal, we performed nonparametric analyses. Specifically, for continuous variables (maternal age, paternal age, parity, maternal body mass index, gestational age, birth weight) we performed Spearman rank correlation, and for binary variables (maternal smoking during pregnancy, newborn gender) we performed Wilcoxon rank sum analysis. Maternal BMI was based on the mother's height and her report of prepregnancy weight at the time of recruitment in the early second trimester of pregnancy. Prior to analysis, regression of birth weight on gestational age, gender, maternal prepregnancy BMI and race was performed, and the residuals used for correlation. A Bonferroni-corrected genome-wide threshold for statistical significance of 1.81 × 10^-6 ^for the relationship between each variable and the levels of DNA methylation at each CpG was employed based on the 27,576 tests performed for each variable. 	Systematic differences between the two races in DNA methylation levels or maternal age are potential confounders in the analyses of correlations between maternal age and newborn DNA methylation. To address the influence of these potential confounders, we used independent genome-wide SNP data collected on a subset (63 African-American, 68 Caucasian) of the participants using the Affymetrix (Santa Clara CA, USA) Genomewide SNP 5.0 and 6.0 arrays. Samples were processed according to Affymetrix's protocol and genotypes were called using the BRLMM (5.0 array) and Birdseed (6.0 array) algorithms with default parameters within the Affymetrix Genotyping Console v4.0 application. Based on the genome-wide patterns of SNP genotypes, loadings on the first two principal components were calculated using the SNP and Variation Suite module of the GoldenHelix v7 software (Golden Helix Inc., Bozeman MT, USA). Based on plots of the loadings on the first two principal components, two genetically distinct groups of participants were identified. Within these comparatively more genetically homogeneous groups, correlations between maternal age and newborn DNA methylation were performed for comparison to analyses across all individuals to determine if population substructuring of genetic variation confounded the main analyses of parental age effects on DNA methylation patterns.

### Functional and disease clustering of significant genes

The Database for Annotation, Visualization, and Integrated Discovery [DAVID, 41, 48] was used to group genes according to functional category and to identify disease processes highly represented among the loci most strongly associated with maternal age. The Bonferroni correction we used to assign significance to loci is likely to be too stringent and to exclude many loci truly correlated with maternal age. Therefore, for the purpose of identifying potentially affected physiological functions and disease processes, we based our selection of loci on false discovery rate q values, or the expected proportion of loci at that threshold of significance that are likely to be false positives. A visual inspection of plots of the number of tests declared significant versus the q value produced by the QVALUE program [49,50] illustrated that the relationship sharply changed beyond a q value of about 0.001. To be conservative, we used a q value threshold of 6.69 × 10^-4 ^(corresponding to a p value of 9.55 × 10^-5^), which results in an expectation that approximately one CpG out of the 1,493 declared significant is a false positive. These 1,493 CpGs belonged to 1,423 different genes. For identification of functional clusters, we used the highest classification stringency and restricted attention to enrichment scores ≥ 1.3, roughly corresponding to a p value of 0.05 (10^-1.3^). As the background reference set of genes, we used the non-redundant list of genes represented on the Humanmethylation27 array as identified by the Entrez gene ID numbers provided by Illumina in their annotation of the array. The identification of imprinted loci was based on the online imprinted gene and parent-of-origin effect database http://igc.otago.ac.nz/home.html[51]. Using liberal criteria, we considered 59 loci with known or polymorphic imprinting patterns or provisional evidence of imprinting.

### Univariate and multivariate analyses of genes whose expression or DNA methylation are characteristic of blood cell types

Previous observations of a relationship between adult age and changes in DNA methylation have sometimes been attributed to changes in blood cell composition as an individual ages. Palmer et al. [38] identified genes whose expression is characteristic of B-cells, T-cell subtypes, granulocytes and lymphocytes. Although they did not directly determine the blood cell type composition of their ovarian cancer case-control cohort, Teschendorff et al. [52] used Palmer et al.'s [38] blood cell subtype-specific expression signatures and the same methylation array used in our study to infer that age-related changes in DNA methylation in their cohort could principally be explained by changes in blood cell composition based on the over-representation of genes whose expression levels are signatures of blood cell subtypes. To indirectly address the possibility that the patterns we observe could be due to maternal age-related changes in the relative proportions of newborn cord blood cell types, we also used the data of Palmer et al. [38].

Based on gene symbols, we identified the overlap between the signature genes reported by Palmer et al. and the genes targeted by the Illumina methylation array. Using all of the probes among these genes, we performed principal components analysis. To visually examine the relationship, we plotted the first principal component versus maternal age. Additionally, we calculated the Spearman rank correlation between maternal age and the first principal component. The principal component analysis summarizes the variability due to all of the probes in the blood cell type signature genes. However, any relationship between maternal age and changes in cord blood cell type composition may be due to a subset of genes or probes. Therefore, we also examined the rank correlation between maternal age and each probe in signature genes.

## List of abbreviations

DAVID: Database for Annotation, Visualization, and Integrated Discovery; DMR: differentially methylated region; OMIM: Online Mendelian Inheritance in Man; CANDLE: Conditions Affecting Neurocognitive Development and Learning in Early Childhood;

## Authors' contributions

RMA designed the project and directed the collection and analyses of experimental data. FAT assisted in the interpretation of the data and directs the CANDLE project, its experimental design, and collection of biological samples. FT and JK participated in the analyses and interpretation of the data. All authors read and approved the final manuscript.

## Pre-publication history

The pre-publication history for this paper can be accessed here:

http://www.biomedcentral.com/1471-2350/12/47/prepub

## Supplementary Material

Additional file 1 **Table S1**. Summary of statistical results for each CpG, including select annotation from Illumina. Click here for file

Additional file 2 **Table S2**. List of the 144 CpGs with genome-wide significant correlation between newborn methylation and maternal age and the CpG with next smallest p value. Whether the 1^st ^and 2^nd ^CpG exhibit correlation with maternal age in the same direction and whether the 2^nd ^CpG is significant at a nominal p value of 0.05 is indicated. Click here for file

Additional file 3 **Table S3**. Output from DAVID analysis of disease clustering among the 1,423 genes whose level of methylation was correlated with maternal age at a q value of 6.69 × 10^-4^, corresponding to an expectation of one false positive and p = 9.55 × 10^-5^. Click here for file

Additional file 4 **Table S4**. Output of functional clustering of 1,423 genes whose level of newborn methylation was correlated with maternal age at a q value of 6.69 × 10^-4^, corresponding to an expectation of one false positive and p = 9.55 × 10^-5 ^by DAVID. Click here for file

Additional file 5 **Table S5**. List of 1,273 Humanmethylation27 array probes and 658 genes overlapping with blood cell type signature genes identified by Palmer et al. (2006. Cell-type specific gene expression profiles of leukocytes in human peripheral blood. BMC Genomics 7, 115). Click here for file
